# Towards High-performance Materials Based on Carbohydrate-Derived Polyamide Blends

**DOI:** 10.3390/polym11030413

**Published:** 2019-03-04

**Authors:** Aleksandra A. Wróblewska, Nils Leoné, Stefaan M. A. De Wildeman, Katrien V. Bernaerts

**Affiliations:** Faculty of Science and Engineering, Biobased Materials, Maastricht University, P.O. Box 616, 6200 MD Maastricht, The Netherlands; olaelwroblo@gmail.com (A.A.W.); nils.leone@maastrichtuniversity.nl (N.L.); s.dewildeman@maastrichtuniversity.nl (S.M.A.D.W.)

**Keywords:** biobased polyamides, blending, high *T_g_*, mechanical properties

## Abstract

A bio-derived monomer called 2,3:4,5-di-*O*-isopropylidene-galactarate acid/ester (GalXMe) has great potential in polymer production. The unique properties of this molecule, such as its rigidity and bulkiness, contribute to the good thermal properties and appealing transparency of the material. The main problem, however, is that like other biobased materials, the polymers derived thereof are very brittle. In this study, we report on the melt blending of GalXMe polyamides (PAs) with different commercial PA grades using extrusion as well as blend characterization. Biobased PA blends showed limited to no miscibility with other polyamides. However, their incorporation resulted in strong materials with high Young moduli. The increase in modulus of the prepared GalXMe blends with commercial PAs ranged from up to 75% for blends with aliphatic polyamide composed of 1,6-diaminohexane and 1,12-dodecanedioic acid PA(6,12) to up to 82% for blends with cycloaliphatic polyamide composed of 4,4′-methylenebis(cyclohexylamine) and 1,12-dodecanedioic acid PA(PACM,12). Investigation into the mechanism of blending revealed that for some polyamides a transamidation reaction improved the blend compatibility. The thermal stability of the biobased PAs depended on which diamine was used. Polymers with aliphatic/aromatic or alicyclic diamines showed no degradation, whereas with fully aromatic diamines such as p-phenylenediamine, some degradation processes were observed under extrusion conditions (260/270 °C).

## 1. Introduction

**Figure 1:**
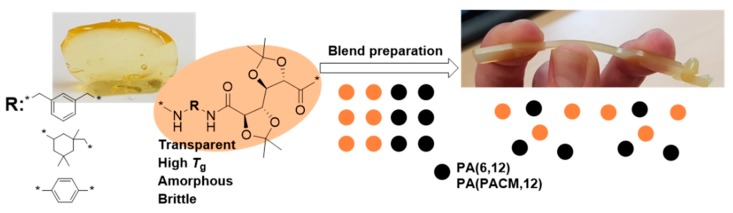
Approach for the preparation of blends from biobased GalX polyamides (PAs). PA(6,12)—polyamide composed of 1,6-diaminohexane and 1,12-dodecanedioic acid, PA(PACM,12) composed of 4,4′-methylenebis(cyclohexylamine) and 1,12-dodecanedioic acid.

Carbohydrate-based monomers constitute a very promising replacement for fossil-based building blocks by providing a biobased alternative to some commonly used monomers [1,2,3,4], and by offering new functionalities [5,6]. The acetal of galactaric acid (GalX) is used for polyamide synthesis and is obtained from sugar beet pulp, a second-generation feedstock which does not compete with the food industry [7,8]. As a monomer, GalX possesses the advantage of a rigid and bulky structure which, upon incorporation into polyamides, can result in polyamides with high *T_g_* [9]. On the downside, however, like other carbohydrate-based polymers, these polyamides have the tendency to be brittle at room temperature (Figure 1) [1,10]. Similarly, as for biobased poly(lactic acid) (PLA), the mechanical performance of these polymers needs to be further improved. PLA is an extensively researched biobased polyester with high potential for wide-spread applications. However, it possesses poor ductility, with an elongation at break of only around 3% [11,12,13].

There are several ways to improve the performance of biobased materials: copolymerization [14,15,16,17,18], blending and/or reactive blending [11,19,20,21,22,23], or by modification through the addition of additives (e.g., plasticizers or impact modifiers) [24,25]. Furthermore, high-modulus polyamide materials can be developed using sustainable processing like electron-induced reactive processing [26,27] and also by creating nano-phase rubber domains in the polyamide matrix [28].

The most common combination for carbohydrate-based polymers is the synthesis of copolyesters of biacetalized galactaric acid derivatives with aromatic and aliphatic diols [14,15,17,29,30,31,32,33,34], and, more recently, GalX (co)polyamides [9,35,36]. The conducted research demonstrates that the successful copolymerization gives polyesters with improved properties (i.e., higher elongation at break) than pure carbohydrate-based polymers themselves [15].

Another approach to increasing the performance of polymer materials is blending and/or reactive blending. In general, blending has numerous advantages over other modification methods, as it is considered to be cheap and industrially relevant. However, in the case of blending, the miscibility of the two components plays a predominant role in obtaining blends with improved mechanical properties compared to the separate homopolymers [37,38].

The blending of natural biopolymers such as starch, cellulose, proteins, chitin/chitosan or lignin with other (bio) polymers has been proven to be an interesting method to obtain new biomaterials with enhanced properties to meet the requirements of specific applications [39,40]. Another abundant class of biopolymers are aliphatic polyesters, such as PLA and poly(hydroxyalkanoate) (PHA), although they suffer from brittleness [41]. While blending with polyamides, for example, has proved to be an effective way to overcome the brittleness of PLA [42,43,44] and PHB [45], no reports are known on the improvement of the mechanical properties of another class of brittle biopolymers—namely, GalX-containing polyamides. 

Facing this lack of relevant scientific data, we want to initiate the research concerning the improvement of the properties of carbohydrate-based synthetic polymers, which could form the basis for the material development thereof. Such polymers deserve significant attention as they can offer a wide variety of advantageous properties, such as transparency or thermal properties for material design, but also the introduction of new functionalities [35]. However, as with all polymeric materials at the beginning of their development, broad investigation is required. Therefore, we proposed the melt-blending of GalX-based amorphous homopolymers with two different commercially available polyamides to improve the thermal properties of the latter, and the mechanical properties (i.e., brittleness) of the former. The polymers were mixed in an extruder at elevated temperatures and the properties of the blends were evaluated. 

## 2. Experimental Part

### 2.1. Materials

Trogamid CX9704 (Evonik, Antwerp, Belgium) was purchased from Brenntag (NL) (*M_n_* = 6000 g/mol, *Ð* = 2.20, *T_g_* = 130 °C, viscosity number > 120 cm^3^/g according to ISO 307). Pentafluorophenol-d_6_ 99.0% (PFP-d_6_) and polyamide 6,12 (PA(6,12), *M_n_* = 10,000 g/mol, *Ð* = 1.65, *T*_m_ = 215 °C, viscosity number 95 cm^3^/g according to ISO 307, 1157, 1628) were purchased from Sigma-Aldrich (Zwijndrecht, the Netherlands) and used as supplied. HFIP (1,1,1,3,3,3-hexafluoro-2-propanol) was purchased from Acros Organics (Geel, Belgium). Irgafos 168 and Irganox 1010 were purchased from BASF (Ludwigshafen, Germany). CDCl_3_ was purchased from Cambridge Isotope Laboratories (Andover, MA, USA). 

### 2.2. Methods

The synthesis of poly(1,3-phenylenedimethanamine–*co*-2,3:4,5-di-*O*-isopropylidene-galactaramid) PA(MXD,GalXMe), poly(5-Amino-1,3,3-trimethylcyclohexanemethanamine-*co*-2,3:4,5-di-*O*-isopropylidene-galactaramid) PA(IPDA,GalXMe) and poly(paraphenylene-2,3:4,5-*co*-di-*O*-isopropylidene-galactaramid) PA(PPDA,GalXMe) was conducted according procedures described by Wróblewska et al. [9]. 

#### A Typical Procedure for Blend Preparation

PA(MXD,GalXMe) (1.5 g, 50 wt%), PA(6,12) (1.5 g, 50 wt%) and antioxidants Irganox 1010:Irgafos 168 at a ratio of 1:2 (0.003 g, 0.1 wt%) were fed into an Xplore MC5 twin-screw mini-extruder under nitrogen atmosphere set with three different temperature zones 270 °C (top), 260 °C (middle), and 260 °C (bottom) and a screw speed of 100 RPM. Polymers were mixed for 1 min followed by the evacuation of the chamber to the injection molding hose at 270 °C. The mold temperature was set to 50 °C to obtain dog-bone-shaped specimens. Injection molding was performed with an Xplore IM5.5

### 2.3. Characterization

The molecular weight of polyamides was determined via gel permeation chromatography (GPC). The polymers were dissolved in 1,1,1,3,3,3-hexafluoroisopropanol (HFIP) with 0.019% NaTFA salt. The sample for GPC measurement was prepared by dissolving 5.0 mg of the polymer in 1.5 mL of the solvent. The solutions were filtered over a 0.2 μm PTFE syringe filter before injection. The GPC apparatus was calibrated with poly(methyl methacrylate) standards. Two PFG combination medium microcolumns with 7 µm particle size (4.6 × 250 mm, separation range 100–1,000,000 Da) and a precolumn PFG combination medium with 7 µm particle size (4.6 × 30 mm) were used in combination with a refractive index (RI) detector in order to determine molecular weight and dispersities. 

The *1D NMR* spectra were recorded on a Bruker Avance 500 MHz in a mixture of CDCl_3_ and PFP-d_6_. 

The 2D-HMBC spectra were recorded on a Bruker 500 MHz using the standard Bruker pulse program hmbcgpl2ndqf with the following parameters: acquisition TD 4096 (f2) ×128 (f1), SW = 12.0 ppm (F2), 180 ppm (F1); O1, 12,558.27 Hz; O2, 3007.48 Hz; D1 = 1.50 s; CNST6 = 135 CNST7 = 155; acquisition time, F2 channel, 340.787 ms; F1 channel, 2.827 ms; processing, SI = 1024 (F2, F1), WDW = SINE.

Differential scanning calorimetry (DSC) curves were recorded on a Netzsch Polyma 2014 DSC. DSC data were obtained from about 5 mg of polymer/blend at heating/cooling rates of 10 °C min^−1^ under a nitrogen flow of 20 mL min^−1^. Indium, zinc, tin, and bismuth were used as standards for temperature and enthalpy calibration. DSC heating and cooling cycles were performed from 25 °C to 250–300 °C. The melting temperatures (peak maximum) and the enthalpy values reported correspond to the second heating cycle. Glass transition temperatures are reported as midpoint values for the second heating cycle.

Scanning electron microscopy (SEM) analysis of the freeze-fractured samples was done with a PhenomPro apparatus equipped with cold cathode field-emission source and backscattered electron detector (BSD) with optical microscopy magnification up to 135× and electron magnification of 80–15,000× with an acceleration voltage of 5 kV. The samples were sputter-coated for 4 min with Au using a Cressington Sputter Coater 108Auto.

Mechanical properties of polymer blends were characterized by performing tensile strength tests, with five replicates of each. Before performing the mechanical tests, the polyamide specimens were dried for 24 h at 80 °C. Tensile tests were performed with a Zwick/Roell Z020 tensile tester equipped with a 10 kN load cell. The tests were performed on injection-molded samples having dimensions 75 mm × 4 mm × 2 mm. A grip-to-grip separation of 50 mm was used. The samples were loaded with a constant crosshead speed of 25 mm·min^−1^. Rheology was done on an Anton Paar MRC702 TwinDrive Rheometer with a CTD450 convection heating system. The frequency sweep was recorded every 20 °C starting from 160 up to 240 °C and reduced to the reference temperature of 220 °C.

## 3. Results and Discussion

### 3.1. Preparation of Blends

**Figure 2:**
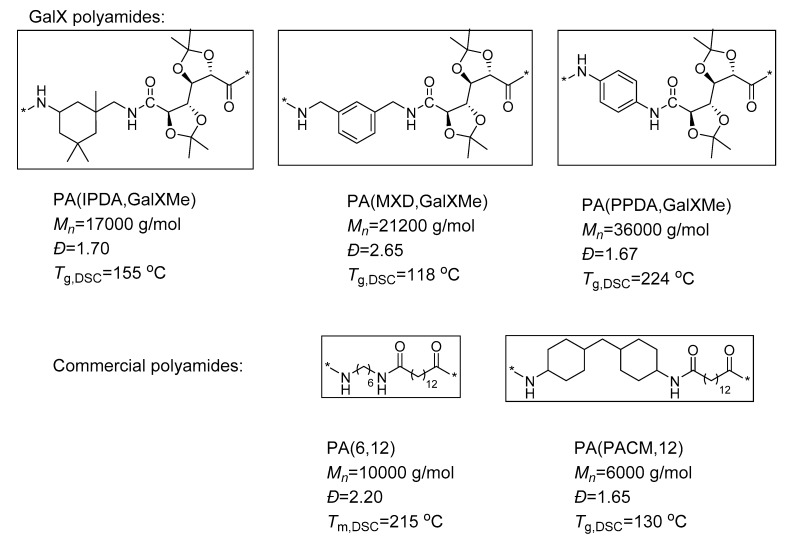
Structures and molecular weight data of polyamides composed of acetals of galactarate (GalX) and diamines (PA(IPDA,GalXMe), (PA(MXD,GalXMe), and PA(PPDA,GalXMe)) as well as commercial polyamides ((PA(6,12) and PA(PACM,12)) that were chosen for the blending experiments.

Three GalX polymers were chosen to be blended with commercial polyamides, namely semicrystalline PA(6,12) and Trogamid CX9704. The latter is an amorphous, transparent polyamide with high glass transition temperature composed of PACM and 1,12-dodecanedioic acid. All GalX polymers are amorphous, transparent materials and the chemical characteristics of the used polymers are summarized in Figure 2. The GalX PAs chosen for the blending experiments differed with respect to the incorporated diamine: alicyclic, partially aromatic, and aromatic diamine (Figure 2). The list of the prepared blends is presented in Table S1.

**Figure 3:**
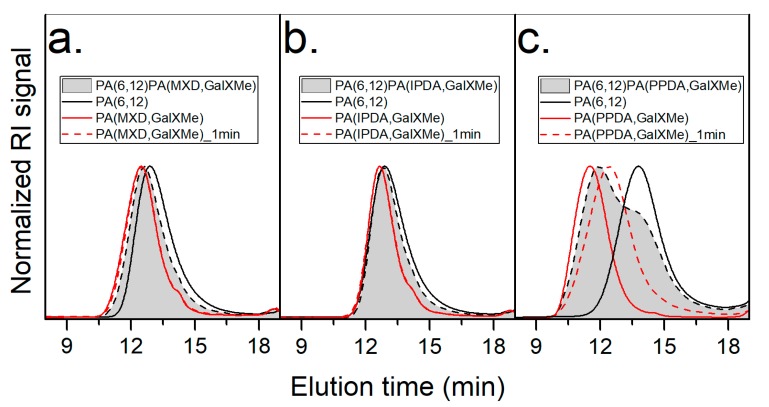
Overlay of the gel permeation chromatography (GPC) traces of the two homopolymers (GalXMe PA ___ and PA(6,12) ___), homopolymer of GalXMe after 1 min of extrusion at 260/270 °C (---), and blend of the corresponding homopolymers after 1 min of extrusion at 260/270 °C (**grey**) (**a**) PA(MXD,GalXMe), (**b**) PA(IPDA,GalXMe), and (**c**) PA(PPDA,GalXMe) blend. RI: refractive index.

Blends were prepared using a twin-screw mini-extruder. Polymers with alicyclic and partially aromatic diamines were easily processable at temperatures above 260 °C even for an elongated time, as confirmed by GPC analysis (Figure 3a,b). Polyamides made using the aromatic diamine PPDA, however, visibly degraded during extrusion at 260/270 °C. In order to evaluate the thermal stability of pure PA(PPDA,GalXMe) polyamide, the polymer was fed into the extruder chamber at 260/270 °C and mixed for 1 min. After this time the chamber was evacuated, and the obtained polymer was in the form of black-brownish rods. The GPC trace of this polymer showed that the whole distribution shifted towards lower molecular weight values (Figure 3c). This was a decisive factor in the determination of the extruding time, which was eventually limited to 1 min.

The molecular weight distribution of the blend was bimodal when there was a big difference in the molecular weight of the homopolymer components (Figure 3c) or became the average of the two distributions when the difference between the molecular weight of the single components was small (Figure 3a,b). The latter case was applicable for PA(MXD,GalXMe) and PA(IPDA,GalXMe) blends with PA(6,12) for which the difference in molecular weight was only between 200 and 1000 g/mol. The distribution of the PA(6,12)_50_PA(PPDA,GalXMe)_50_ blend was bimodal with the remark that the position of the peak maximum of the PA(6,12) part did not change, in contrast to the PA(PPDA,GalXMe) peak which shifted towards lower molecular weight values. Blending with other polymers suppressed degradation processes, which was seen as an improvement of the appearance of the PA(6,12)_50_PA(PPDA,GalXMe)_50_ blend when compared to pure PA(PPDA,GalXMe) homopolymer after thermal processing and as measured by GPC. Lastly, no discoloration or otherwise-manifested degradation was observed regarding PA(IPDA,GalXMe) and PA(MXD,GalXMe) polymers during the extrusion and blending experiments.

### 3.2. Thermal and Morphological Characterization of the Blends

#### 3.2.1. Thermal Properties of PA(6,12) Blends

**Figure 4:**
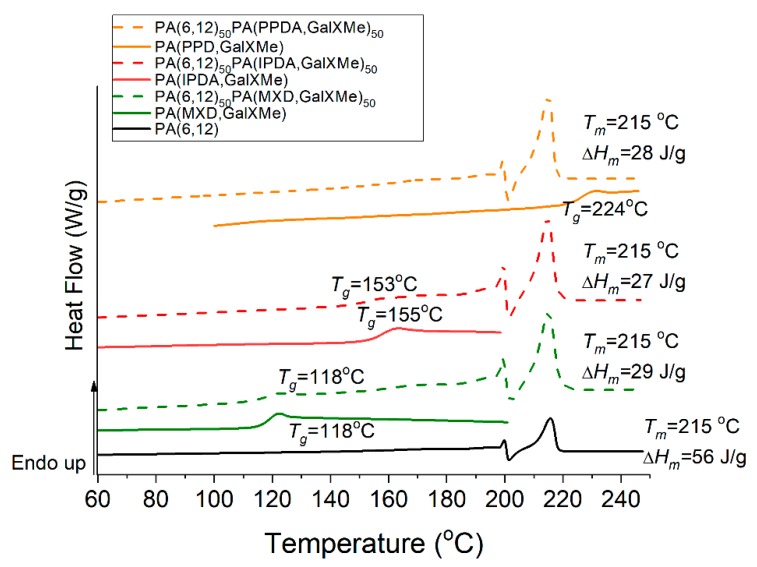
Differential scanning calorimetry (DSC) curves for homopolyamides and PA(6,12) blends representing heat flow (W/J) vs. temperature with endothermic exchange up.

Figure 4 represents the DSC results recorded for homopolymers and their blends. GalX homopolymers had *T*_g_ values of 118 °C for PA(MXD,GalXMe), 155 °C for PA(IPDA,GalXMe) and 224 °C for PA(PPDA,GalXMe). For PA(MXD,GalXMe) and PA(IPDA,GalXMe) blends with PA(6,12) the *T*_g_ for the GalX component was clearly distinguishable, indicating incompatible blends. Only one *T_g_* is observed for miscible systems, and it is generally between the *T*_g_s of the pure components. Partial miscibility can be recognized by a shift of the *T*_g_s compared to the parent polymers. A partially miscible system exhibits two *T*_g_s that are slightly shifted from that of the neat components. An immiscible system exhibits two *T*_g_s but exactly that of the neat components [46]. For the blend with PA(PPDA,GalXMe) no glass transition corresponding to the GalX component could be discerned because it overlapped with the melting point of the aliphatic PA(6,12). Since only the amorphous part in highly crystalline aliphatic polyamides like PA(6,12) contributes to the glass transition, its *T*_g_ could not be detected under the used DSC conditions.

#### 3.2.2. Thermal Properties of PA(PACM,12) Trogamid CX Blends

**Figure 5:**
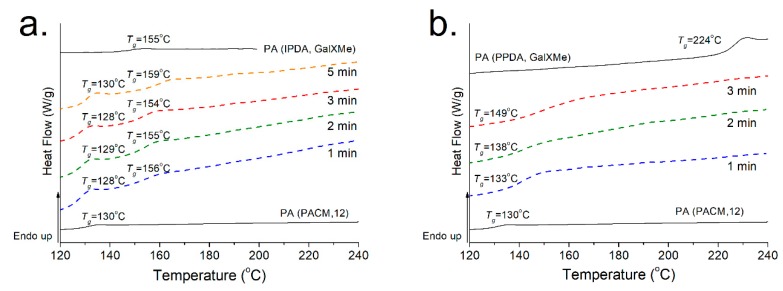
DSC curves for blends of homopolyamides with PA(PACM,12) representing heat flow (W/J) with endothermic exchange up and the variation of the profiles as a function of the processing time. (**a**) PA(PACM,12)_50_PA(IPDA,GalXMe)_50_ and (**b**) PA(PACM,12)_50_PA(PPDA,GalXMe)_50._

Subsequently, blends of PA(PACM,12) with GalX PAs were investigated (Figure 5). Such experiments were limited to using only PA(IPDA,GalXMe) and PA(PPDA,GalXMe) since we wanted to focus on the improvement of the thermal and mechanical properties of the polyamides and PA(MXD,GalXMe) has a lower *T*_g_ than PA(PACM,12). Initially, DSC curves were recorded for polymer samples after one minute of blending at 260/270 °C. The analysis revealed that PA(PACM,C12) was not compatible with PA(IPDA,GalXMe), as shown by the two separate glass transition regions at 130 °C and 155 °C, with the first *T*_g_ corresponding to amorphous Trogamid CX and the second to PA(IPDA,GalXMe) (Figure 5a). Both *T*_g_s were clearly visible, in contrast to blends with PA(6,12). In parallel, DSC curves were recorded for PA(PPDA,GalXMe) with PA(PACM,12) blends, where it was noticed that one *T*_g_ was present at values slightly higher than that for the commercial component (from 130 °C for pure PA(PACM,12) to 133 °C for the blend after one minute of mixing) (Figure 5b), an indication of partial miscibility. This small shift registered for the PA(PPDA,GalXMe) blend stimulated further investigation of the phenomenon. In order to establish whether mixing time influences the miscibility of the components, more samples were prepared for experiments with different mixing times. No influence of time on the quality of mixing was observed for PA(IPDA,GalXMe) polymer blends, which showed the same transitions corresponding to the individual components, regardless of the mixing time. Conversely, PA(PPDA,GalXMe) blended with PA(PACM,12) (Figure 5b) showed a correlation of the transition region with the time of mixing: the longer the mixing was applied, the higher the transition region. After 3 min of mixing, the glass transition of the prepared blend was increased to 149 °C. However, longer mixing times led to the degradation of the PA(PPDA,GalXMe) polymer blend. As a result of degradation after extended mixing with PA(PPDA,GalXMe) (see GPC results in Figure 3) and no influence of time on the miscibility of PA(IPDA,GalXMe) blends, a 1 min blending time was applied for all subsequent experiments.

#### 3.2.3. Morphological Study of the Blends by SEM

**Figure 6:**
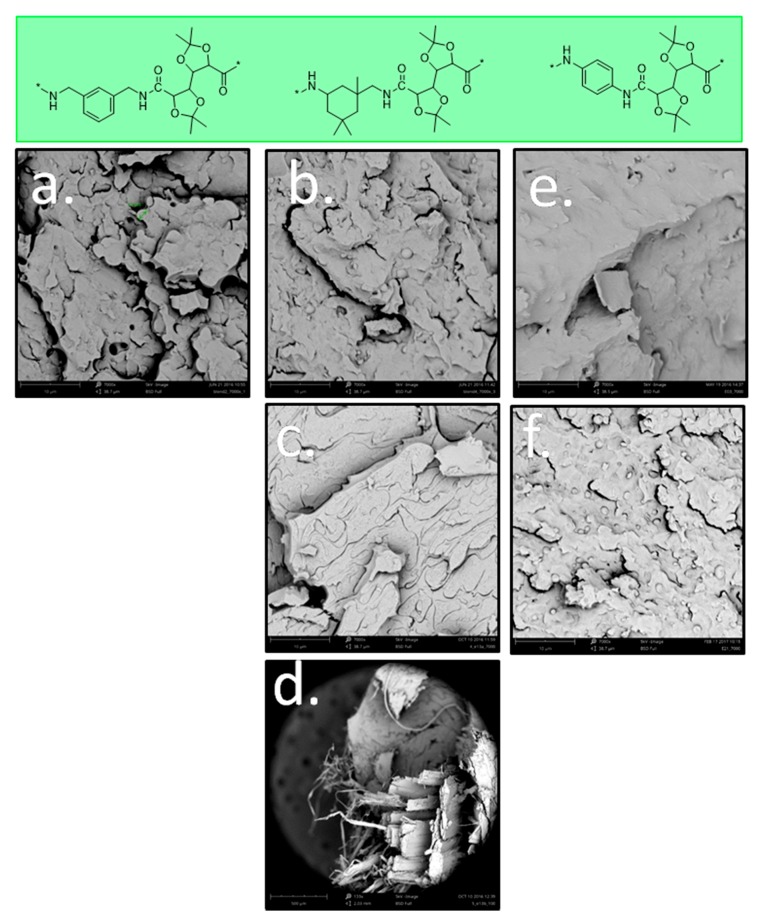
SEM images of blends (**a**) PA(6,12)_50_PA(MXD,GalXMe)_50_ (7000×), (**b**) PA(6,12)_50_PA(IPDA,GalXMe)_50_ (7000×), (**c**) PA(PACM,12)_50_PA(IPDA,GalXMe)_50_ (7000×), (**d**) PA(PACM,12)_50_PA(IPDA,GalXMe)_50_ (133×), (**e**) PA(6,12)_50_PA(PPDA,GalXMe)_50_ (7000×), and (**f**) PA(PACM,12)_50_PA(PPDA,GalXMe)_50_ (7000×).

In principle, in order to achieve a good-quality blend, polymers should be miscible with each other. In practice, however, the blending of biobased polyamides often results in an immiscible blend and requires the addition of a compatibilizer to improve polymer dispersion. SEM images were collected to investigate the miscibility, compatibility, and morphology of blends with PA(6,12) and PA(PACM,12). Figure 6 presents the SEM images of blends with PA(MXD,GalXMe), PA(IPDA,GalXMe), and PA(PPDA,GalXMe). The analysis of microscopic images revealed that blending of PA(MXD,GalXMe) (Figure 6a) and PA(IPDA,GalXMe) (Figure 6b,c) resulted in a two-phase morphology in PA(6,12) or PA(PACM,12). Unlike the aliphatic polyamides, however, PA(PPD,GalXMe) blends (Figure 6e,f) showed partial compatibility, as was also observed by DSC.

An interesting phenomenon was observed after the analysis of PA(PACM,12) blends with PA(IPDA,GalXMe). Under microscopic magnification of 7000× the blend appeared as a two-phase morphology (Figure 6c) but under visual inspection the material showed strong orientation, namely the formation of fibers (Figure 6d). The fibers could not be completely ground even when ground in liquid nitrogen. It is suspected that the formation of fibers originated from strong phase separation that might have been caused by intramolecular hydrogen bond formation between the acetal oxygen of GalXMe motifs and the amide bonds of the GalXMe polyamide. The formation of hydrogen bonds between the amide groups of both polymers in the blend might have been hampered by the bulky structure of both the GalX and the PACM diamine. Furthermore, with diamines that are much less bulky (e.g., PPDA), partial miscibility of the blends was observed as well as the occurrence of transamidation reactions (see further), further confirming the significance of the diamine structure’s bulkiness.

We also investigated how the morphology of PA(PACM,12)_50_PA(IPDA,GalXMe)_50_ blends changed as a function of the extrusion time. DSC curves revealed the indifference of the transition temperatures of those blends towards the extrusion time (Figure 5a). However, SEM results provided insight into a microscopic effect of time. Figure S1 contains three SEM images of the blend after 1, 2, and 5 min of blending. The extrusion time did indeed reduce the domain sizes. However, even after 5 min of blending they were still not compatible. 

#### 3.2.4. Structural Transformations Induced by the Thermal Processing of PA(PPDA,GalXMe) with PA(PACM,12)

**Figure 7:**
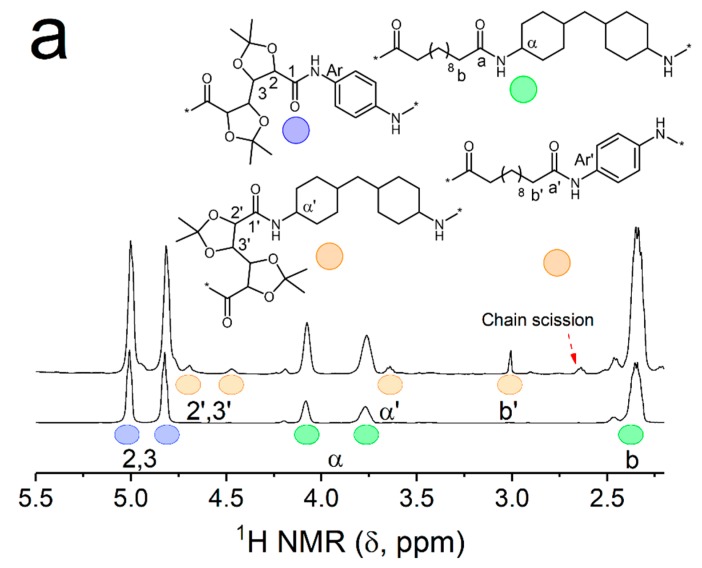

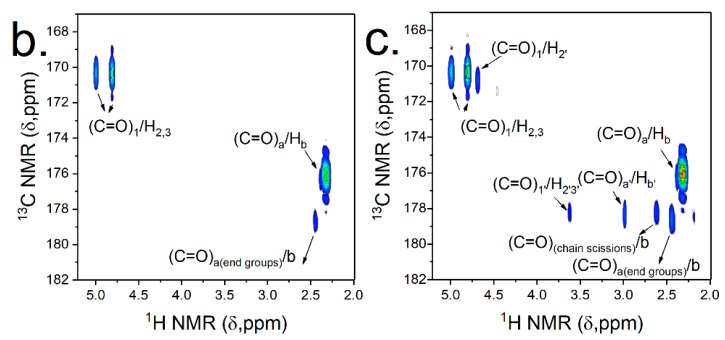
NMR spectra of PA(PPDA,GalXMe)_50_PA(PACM,12)_50_ blends. (**a**) ^1^H NMR spectra of blends prepared in the extruder at 260/270 °C for 3 min (top) and in solution at room temperature (RT; bottom), (**b**) ^1^H-^13^C HMBC spectra of blends prepared in solution at RT and (**c**) ^1^H-^13^C HMBC spectra of blends prepared in the extruder at 260/270 °C for 3 min.

The observed partial compatibility and degradation processes occurring during the blending of PA(PPDA,GalXMe) might consist of different phenomena. The thermal degradation of aromatic amine moieties present in the sample might include the oxidation of amines or imide formation [47,48], but at the same time partial miscibility of the PA(PPDA,GalXMe) blends was observed. This suggests that reactions between the two components might have occurred during blending. To elucidate this, 2D ^1^H-^13^C HMBC correlation NMR spectroscopy through multiple bonds was used. Spectra were recorded for samples prepared by mixing of the two polymers (i.e., PA(PPDA,GalXMe) and PA(PACM,12)) on the one hand at room temperature in a deuterated solvent, and on the other hand after extrusion at 260/270 °C for 3 min. On the ^1^H NMR spectrum (Figure 7a) and on the 2D spectrum (Figure 7b,c) of the blend prepared at room temperature (RT), peaks from two components—PA(PPDA,GalXMe) and PA(PACM,12)—are visible. Peaks correlate as follows: the carbonyl group of PA(PPDA,GalXMe) (C=O)_1_ with protons H_2,3_, the carbonyl group of PA(PACM,12) (C=O)_a_ with protons H_b_ and the carbonyl group of the PA(PACM,C12) end group (C=O)_a(end group)_ with protons H_b_ (Figure 7b). After the extrusion, new peaks were visible (Figure 7a, top and Figure 7c), corresponding to a transamidation reaction between the two polyamides and chain scission. In fact, chain scission might stimulate further degradation processes, which lead to aromatic amine-terminated chains and those end groups are the most susceptible to thermal degradation. The PA(IPDA,GalXMe) spectrum did not show any changes caused by side reactions either before or after extrusion (Figure S2).

### 3.3. Material Properties of Blends

#### Mechanical Performance of Blends

Polyamides derived from carbohydrates are known to be brittle, which in some cases hampers their mechanical properties. In order to evaluate the influence of these components on the mechanical properties of the blends, tensile tests were conducted. The samples were blended for 1 min, and subsequently injection molded, resulting in dog-bone specimens. Table S1 in the Supporting Data contains the compiled results of PA(PACM,12) blends and PA(6,12) blends with different GalX polyamides. Due to the high brittleness of the GalX polyamides, it was not possible to obtain tensile bars with more than 50 wt% of PA(IPDA,GalXMe) and PA(MXD,GalXMe). In all cases, the incorporation of GalX-based polyamides reduced the elongation at break compared to the non-GalX-containing polymer in the blend.

**Figure 8:**
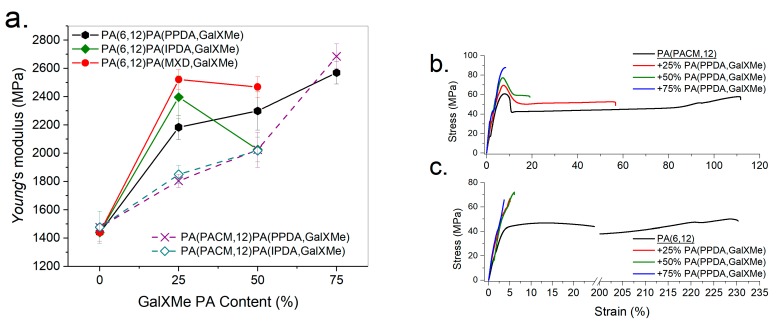
Results of the tensile tests for PA(6,12) and PA(PACM,12) blended with (**a**) PA(PPDA,GalXMe), PA(IPDA,GalXMe), and PA(MXD,GalXMe) expressed as *E* modulus as a function of the amount of GalXMe polyamide and stress–strain curves for (**b**) PA(PACM,12) blended with PA(PPDA,GalXMe) and (**c**) PA(6,12) blended with PA(PPDA,GalXMe).

The mixing of GalX polyamides with PA(6,12) and PA(PACM,12) significantly improved the *E* modulus of the prepared blends (Figure 8a). In general, with a composition of up to 25% of carbohydrate-based polyamide, the Young’s modulus of the blends increased with respect to the pure commercial polyamide. However, attempts to blend PA(6,12) with more than 25% PA(MXD,GalXMe) or PA(IPDA,GalXMe) resulted in a loss of the Young’s modulus and general decay in mechanical performance. It is suspected that this drop was caused by blend incompatibility, which started to dominate the mechanical properties at a higher content of GalXMe polyamide. 

The PA(PPDA,GalXMe) blends outperformed the PA(MXD,GalXMe) and the PA(IPDA,GalXMe) blends, and an increased amount of PA(PPDA,GalXMe) in the blend led to a further increase of the Young’s modulus (up to 82%). As demonstrated in Figure 8, PA(PPDA,GalXMe) was capable of forming blends with the highest GalX content combined with good mechanical properties. Those good mechanical properties can be correlated with the earlier observed partial miscibility of PA(PPDA,GalXMe) blends (DSC, SEM and NMR), in contrast to PA(MXD,GalXMe) and PA(IPDA,GalXMe) blends. Both commercial polyamides PA(PACM,12) and PA(6,12) showed high elongation at break (see Figure 8b,c) with an *E* modulus around 1400 MPa. Upon the addition of PA(PPDA,GalXMe) polyamide, the *E* modulus increased (51%–78% for PA(6,12) blends and 22%–82% for PA(PACM,12) depending on the composition) and the elongation at break decreased.

All blends with PA(6,12) (Figure 8c) could be classified as hard and brittle materials due to a very low elongation and no yielding. However, the elongation at break of PA(PACM,12) did not drop that drastically upon the addition of PA(PPDA,GalXMe) (Figure 8b). Conversely, blends incorporating up to 50% PA(PPDA,GalXMe) yielded upon stretching, with an elongation dependent on the amount of added PA(PPDA,GalXMe). Such blends can therefore be classified as hard and tough materials [49]. 

## 4. Conclusions

Blends from GalXMe polyamides with semicrystalline PA(6,12) and amorphous PA(PACM,12) were successfully prepared by thermal processing in the extruder. The GalXMe polyamides with alicyclic and aliphatic diamines were stable during processing conditions. However, the GalXMe polyamides made with aromatic diamines were susceptible to degradation, particularly under extended processing times.

The blends showed no miscibility with PA(IPDA,GalXMe) and PA(MXD,GalXMe), and partial miscibility with PA(PPDA,GalXMe). PA(PPDA,GalXMe) blends showed no phase separation under the SEM microscope, likely as a result of the compatibilizing effect induced by transamidation reactions occurring during the thermal processing in the extruder. 

While the incorporation of GalXMe polyamides into the composition increased the Young’s modulus up to 82%, the elongation at break decreased, which resulted in a hard and brittle material. On the other hand, the most promising blends with PA(PPDA,GalXMe) showed ductility when they were blended with commercial polyamide PA(PACM,12).

## Data Availability

The raw and the processed data required to reproduce these findings are available from the corresponding author upon request.

## Supplementary Materials

The following are available online at https://www.mdpi.com/2073-4360/11/3/413/s1.
