# Supplementary material for: Towards High-performance Materials Based on Carbohydrate-Derived Polyamide Blends
Source: Polymers (Basel). 2019 Mar 4;11(3):413. doi: 10.3390/polym11030413 (PMC6473389; doi:10.3390/polym11030413)
Supplement: Supplementary file 1 [file polymers-11-00413-s001.pdf]

Supplementary data for

Towards high performance materials based on carbohydrate-derived polyamide blends

*Aleksandra A. Wróblewska, Nils Leoné, Stefaan M.A. De Wildeman, Katrien V. Bernaerts**

Faculty of Science and Engineering, Biobased Materials, Maastricht University,

P.O. Box 616, 6200MD Maastricht, the Netherlands

Table S-1 List of the prepared experiments together with *E* modulus strain at break and tensile strength values. Missing results (-) due to too high rigidity of the material to obtain specimens for test.

Entry*	*E* (MPa)	*E* increase[a] (%)	Strain at break (%)	Tensile strength (MPa)
PA(6,12)	1440 ± 60		237 ± 30	52 ± 3.7
PA(MXD,GalXMe)	-		-	
$PA(6,12)_{75}PA(MXD,GalXMe)_{25}$	2520 ± 70	75	2 ± 0.8	47 ± 15
$PA(6,12)_{50}PA(MXD,GalXMe)_{50}$	2469 ± 73	71	3 ± 0.8	45 ± 5.7
$PA(6,12)_{25}PA(MXD,GalXMe)_{75}$	-		-	-
PA(IPDA,GalXMe)	-		-	-
$PA(6,12)_{75}PA(IPDA,GalXMe)_{25}$	2400 ± 151	67	3 ± 0.7	51 ± 6.0
$PA(6,12)_{50}PA(IPDA,GalXMe)_{50}$	2030 ± 88	41	3 ± 0.1	34 ± 1.3
$PA(6,12)_{25}PA(IPDA,GalXMe)_{75}$	-		-	-
PA(PPDA,GalXMe)	-		-	-
$PA(6,12)_{75}PA(PPDA,GalXMe)_{25}$	2180 ± 86	51	4 ± 0.8	64 ± 2.4
$PA(6,12)_{50}PA(PPDA,GalXMe)_{50}$	2300 ± 135	59	6 ± 0.7	74 ± 2.9
$PA(6,12)_{25}PA(PPDA,GalXMe)_{75}$	2570 ± 80	78	3 ± 0.5	61 ± 7.2
PA(PACM,12)	1480 ± 114		110 ± 4.9	61 ± 0.3
$PA(PACM,12)_{75}PA(IPDA,GalXMe)_{25}$	1850 ± 64	25	5 ± 0.8	64 ± 1.1
$PA(PACM,12)_{50}PA(IPDA,GalXMe)_{50}$	2020 ± 22	36	4 ± 0.3	55 ± 0.9
$PA(PACM,12)_{25}PA(IPDA,GalXMe)_{75}$	-		-	-
$PA(PACM,12)_{75}PA(PPDA,GalXMe)_{25}$	1800 ± 47	22	56 ± 2.2	69 ± 0.8
$PA(PACM,12)_{50}PA(PPDA,GalXMe)_{50}$	2030 ± 124	37	29 ± 17	74 ±3.2
$PA(PACM,12)_{25}PA(PPDA,GalXMe)_{75}$	2690 ± 88	82	6 ± 2.4	76 ± 9.5

* subscripts represent weight %

[a]The value represents an increase of *E* modulus in comparison to *E* modulus of pure commercial polyamide

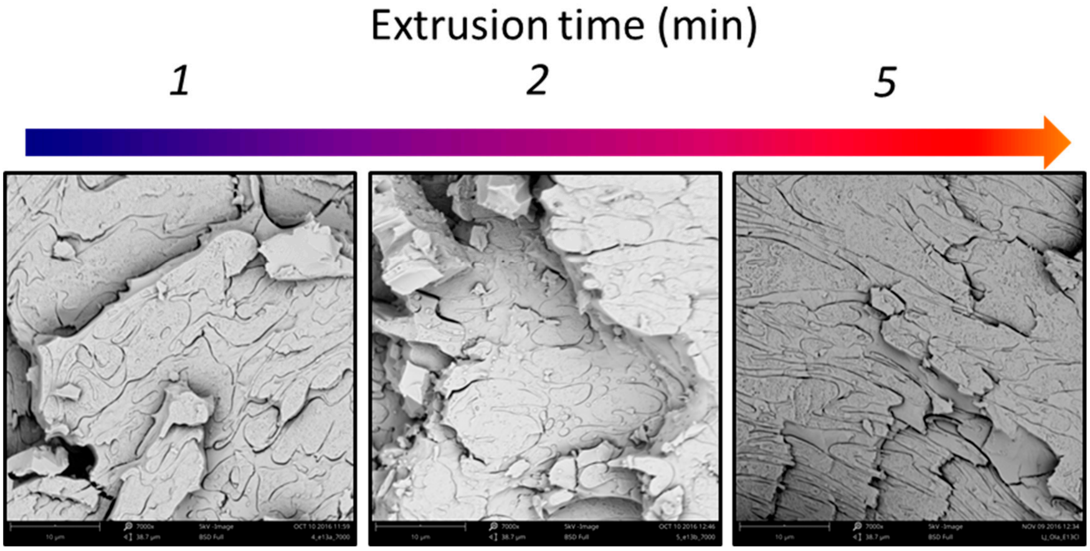

Figure S1. SEM images of $PA(PACM,12)_{50}PA(IPDA,GalXMe)_{50}$ blend after 1, 2 and 5 minutes of blending in the extruder at 260/270 °C.

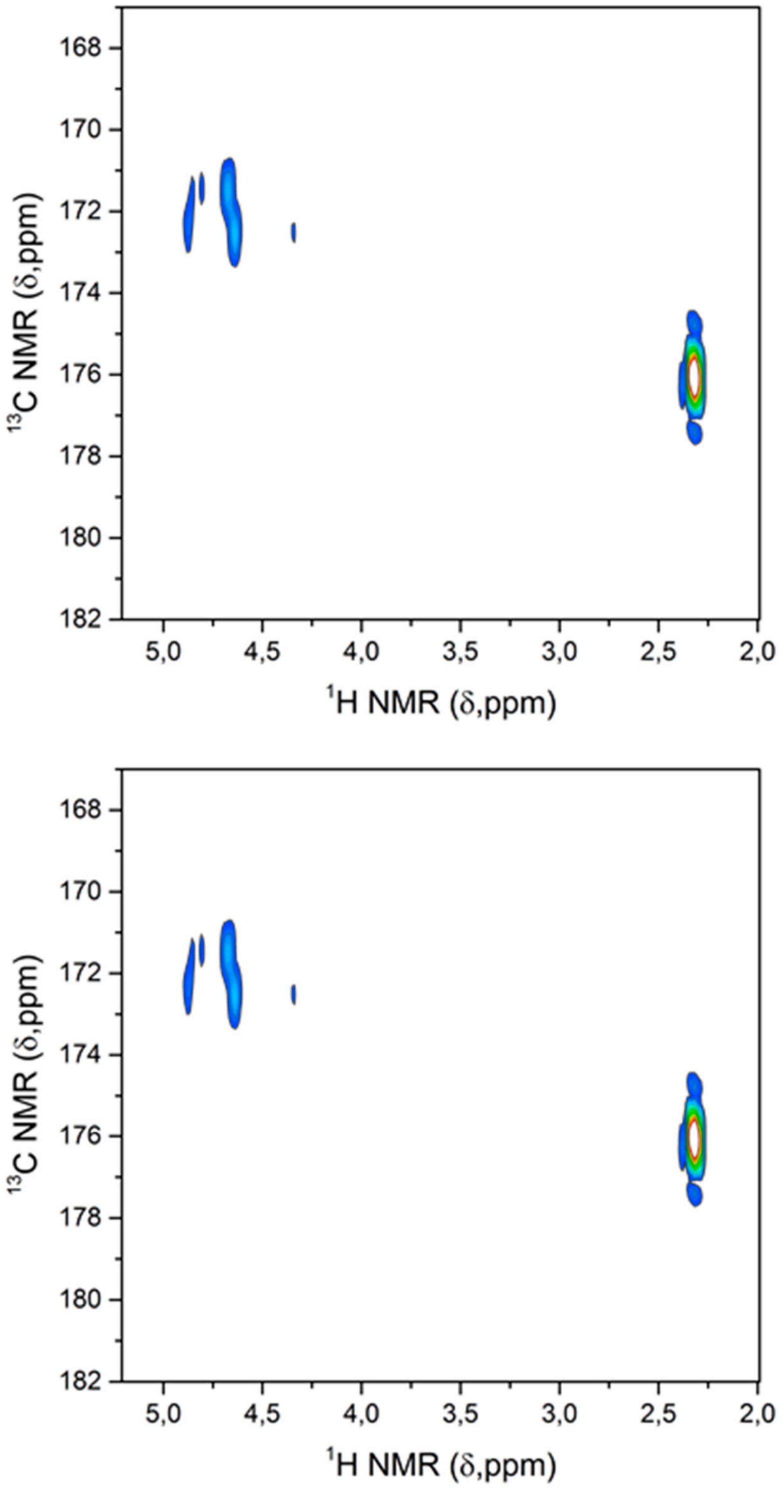

Figure S2. The ^{1}H-^{13}C HMBC of PA(PACM,12)$_{50}$PA(IPDA,GalXMe)$_{50}$ blends prepared in the extruder at 260 °C for 5 min (top) and in solution at room temperature (bottom).
